# Dairy consumption has a partial inverse association with systolic blood pressure and hypertension in populations with high salt and low dairy diets: cross-sectional data analysis from the Iwaki Health Promotion Project

**DOI:** 10.1038/s41440-024-02088-6

**Published:** 2025-01-22

**Authors:** Daisuke Kawata, Hiroshi M. Ueno, Ayatake Nakano, Yota Tatara, Yoshinori Tamada, Tatsuya Mikami, Koichi Murashita, Shigeyuki Nakaji, Ken Itoh

**Affiliations:** 1 Department of Precision Nutrition for Dairy Foods, Hirosaki University Graduate School of Medicine, Hirosaki, Japan; 2 Milk Science Research Institute, Megmilk Snow Brand Co., Ltd., Kawagoe, Japan; 3 Research Center for Health-Medical Data Science, Hirosaki University Graduate School of Medicine, Hirosaki, Japan; 4 Innovation Center for Health Promotion, Hirosaki University Graduate School of Medicine, Hirosaki, Japan; 5 Research Institute of Health Innovation, Hirosaki University, Hirosaki, Japan; 6 Biomedical Research Center, Hirosaki University Graduate School of Medicine, Hirosaki, Japan

**Keywords:** Blood pressure, Dairy consumption, Phosphorus metabolism, Iwaki Health Promotion Project

## Abstract

The prevalence of hypertension in Japan remains high, owing to the high salt content of the typical Japanese diet. Dairy-based foods may reduce blood pressure and hypertension risk. However, dairy consumption is low in Japan, and the relationships between dairy intake and blood pressure or the mechanisms by which dairy products affect blood pressure are not fully understood. This cross-sectional study was conducted as part of the Iwaki Health Promotion Project in Aomori, Japan. A total of 1071 participants were included from those who underwent annual medical checkups in June 2015. Adjusted multivariate linear and logistic regression analyses were performed to analyze the relationships between dairy consumption and blood pressure or hypertension risk. Comprehensive blood biomarker measurements were also performed. Whole- and high-fat dairy consumption was found to have significant inverse associations with systolic blood pressure (SBP) for all participants (*β* = –0.0213, *P* = 0.044) and with SBP and systolic hypertension risk for non-users of antihypertensive medicines (*β* = –0.0306, *P* = 0.011; and OR = 0.9927, *P* = 0.016; respectively). Three blood biomarkers related to phosphorus metabolism (inorganic phosphorus, intact parathyroid hormone, and interleukin-6) were associated with both dairy consumption and SBP. Dairy consumption had a partial inverse association with SBP and hypertension risk in a Japanese population with high salt and low dairy consumption. Analysis of blood biomarkers suggested that phosphorus metabolism is involved in the associations between dairy consumption and blood pressure.

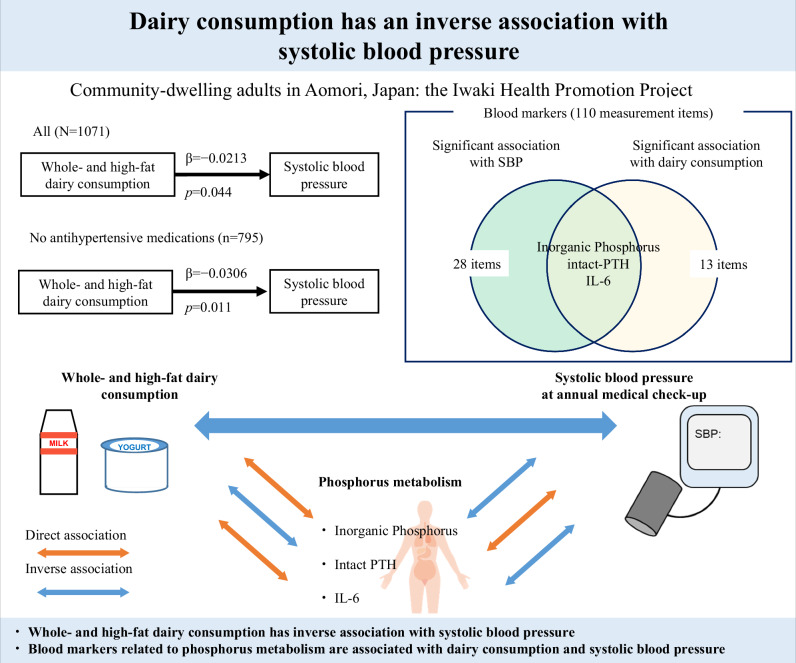

## Introduction

Reducing blood pressure and preventing hypertension are crucial steps toward prolonging life expectancy. High salt intake is a known contributor to hypertension [1, 2], and many Japanese diets are typically high in salt [3, 4]. This high-salt diet can elevate their risk of hypertension, posing challenges in preventing hypertension [5].

Dairy foods have shown promise in reducing hypertension, as some studies indicate an inverse association between blood pressure and dairy consumption [6–8]. Meta-analyses of cohort studies suggest that dairy consumption is either neutrally or beneficially associated with cardiovascular disease [9, 10]. Dairy products are rich in bioactive proteins and essential minerals, including potassium, calcium, and phosphorus [11]. Potassium and calcium are particularly important in the dietary management of blood pressure and hypertension, with phosphorus also emerging as a potential factor [12–14]. Furthermore, minerals derived from milk have been associated with lower blood pressure levels [15]. Additionally, peptides derived from milk proteins have demonstrated antihypertensive effects [15, 16]. However, significant associations were not observed in some studies, including one Mendelian randomization study [17, 18]. A systematic review also found no significant association between total dietary phosphorus intake and blood pressure [19]. As a result, the effects of dairy products on blood pressure yield inconsistent findings.

Additionally, variations in dietary patterns across different regions may contribute to these inconsistencies. In Japan, dairy consumption is ~110 g/day, which is relatively low compared to that in most Western countries [11]. A low intake of dairy products, and dietary patterns that include high salt intake, may be related to the high prevalence of hypertension in Japan. To explore the effects of dairy products on blood pressure, population-based differences in dairy intake and dietary patterns should be considered. In addition, the mechanism underlying the association between dairy consumption and blood pressure remains not fully understood because most studies on the subject have lacked data regarding blood biomarkers. Blood biomarkers can help explain bodily changes and provide clues to elucidate the mechanisms underlying the antihypertensive effects of dairy products. Comprehensive measurements of nontargeted metabolites in biological fluids offer a data-driven approach to assessing dietary exposure without prior knowledge of specific food and nutrition biomarkers [20, 21]. This objective strategy can identify new metabolites and their association with dietary and nutritional status.

The super-multiitem health database from the community-based annual medical checkups in the Iwaki Health Promotion Project (IHPP) [22, 23] enables us to conduct an objective, data-driven exploration of potential biomarkers related to dairy exposure and blood pressure. By analyzing the data from IHPP, this study aimed to clarify the effect of dairy consumption on blood pressure in populations with high salt and low dairy intake, such as the Japanese population, and to explore the potential underlying mechanisms by means of data-driven statistical approaches.

Point of view

- Clinical relevance:
  Dairy intake may suppress the increase in systolic blood pressure and prevent hypertension.
- Future direction:
  Longitudinal studies and intervention trials are warranted to clarify the causal relationship between dairy intake and blood pressure and the involvement of phosphorus intake.
- Consideration for the Asian population:
  From the point of preventing hypertension, dairy intake is recommended for Asians, who consume less dairy products and more salt compared to Westerners.

## Methods

### Ethics approval

This study was conducted in accordance with the 1964 Helsinki Declaration and its later amendments, was approved by the Internal Review Board of Hirosaki University (ref.: #2014-377-1 and 2022-123), and was registered in the Japanese Clinical Trials Registry (UMIN000040459). All included participants provided written informed consent at their time of enrollment in the source cohort in June 2015, and the opt-out procedure for this study was completed in March 2023.

### Study participants

This was a secondary cross-sectional analysis of the IHPP cohort conducted in Aomori Prefecture, Tohoku region, Japan [22, 23]. The IHPP is a community-based preventive medicine program that conducts annual medical checkups for ~1000 residents. We utilized the 2015 IHPP data for this study. A total of 1113 healthy adult residents from the Iwaki region (Hirosaki City, Aomori, Japan) were recruited for the IHPP in June 2015. Data from 1071 participants were analyzed, excluding 42 individuals with missing information regarding dairy consumption. Among the 1071 participants, 412 were male and 659 were female, with ages ranging from 20 to 91 years. Missing data were excluded from individual variables in all regression analyses.

In a subsequent analysis focusing on non-users of antihypertensive medications, 795 participants were included after excluding 276 individuals who were taking such medications. Of the 795 participants, 309 were male and 486 were female, with ages also ranging from 20 to 91 years.

### Participant characteristics and blood biomarkers

Age, sex, and body mass index (BMI) represented the participants’ demographic characteristics. Weight and height were measured to calculate BMI. Questionnaires were administered to assess smoking status (current-smoker, former-smoker, or never-smoker), drinking status (current-drinker, former-drinker, or never-drinker), exercise frequency per week, and prescription drug use. SBP and DBP were measured using the Elemano blood pressure monitor H-55 (TERMO, Japan). According to the latest Japanese guidelines regarding hypertension management [24], participants with SBPs of ≥140 or DBPs of ≥90 were classified as having hypertension. Participants with SBPs of ≥140 and those with DBPs of ≥90 were subdivided as having systolic hypertension and diastolic hypertension, respectively. A total of 110 biomarkers were measured in blood samples collected during the participants’ health checkups. The list and measurement methods for the biomarkers are summarized in Supplementary Table 1.

### Food frequency questionnaire

To investigate the participants’ dietary intakes, we used the brief-type self-administered Diet History Questionnaire (BDHQ). The BDHQ is a self-administered questionnaire used to survey dietary status over the month preceding a patient’s medical checkup visit [25, 26]. Validation studies for the BDHQ, which assessed dairy and mineral intakes, utilized 16-day dietary records [25, 27]. For dairy products and calcium, energy-adjusted intake from the food group showed comparable results, with no statistically significant differences between the dietary records and BDHQ [27]. However, some minerals estimated by BDHQ, such as potassium in females and phosphorus in males, differed from the estimates obtained through dietary records [25]. We used energy-adjusted intake of energy, nutrients, and foods (per day and 1000 kcal) for further analyses, using an ad hoc computer algorithm based on the BDHQ validation study [25–27]. Since the BDHQ has two dairy product categories, “low-fat dairy products” and “whole- and high-fat dairy products,” our analyses were performed respectively for low-fat, whole- and high-fat, and total dairy products. Since the BDHQ does not provide the percentage of energy intake for individual nutrients by default, we estimated these percentages for carbohydrates, proteins, and fats using the Atwater system [28].

### Statistical analysis

Group comparisons of the participants with and without hypertension were performed using the Chi-squared test for categorical variables and the Mann-Whitney U test for continuous ones. Group comparisons regarding dairy intake were performed using the Steel-Dwass test. According to the Japanese dietary guidelines, a serving size for dairy products was established at 200 g/day [29].

Adjusted multivariate linear regression analysis was performed to identify the association between dairy consumption and blood pressure. A multivariate logistic regression analysis was performed to analyze the relationship between dairy consumption and hypertension risk. For all of the participants, three models were analyzed. In Model 1, we performed simple adjustments for age, sex, BMI, and the use of antihypertensive medications. Since smoking is a well-established risk factor for cardiovascular diseases and is known to increase blood pressure [30], we included smoking status in Model 2. Furthermore, the Japanese guidelines identify six lifestyle modifications for hypertension management: salt restriction, food intake including vegetables and fruits, maintaining an appropriate body weight, exercise, drinking habits, and smoking status [24]. Therefore, Model 3 incorporated these lifestyle factors, including salt intake, vegetable and fruit consumption, exercise time, and drinking status, along with the adjustments made in Model 2. For those participants not taking antihypertensive medications, the three models were adjusted similarly, minus the inclusion of the antihypertensive medications. Multivariable linear regression analysis was also used for our comprehensive analysis of blood biomarkers, using the same adjustments as Model 3. All available blood biomarkers from the IHPP 2015 database, totaling 110, were included in this analysis. The associations between mineral intake and blood pressure were analyzed using multivariate linear regression, adjusted for age, sex, BMI, use of antihypertensive medications (only for the analysis of all participants), smoking status, salt intake, exercise time, and drinking status.

The analyses were conducted using Python 3.11.4. Statistical significance was set at *P* < 0.05.

## Results

### Background characteristics

In total, 1113 participants were enrolled. After excluding 42 participants with missing BDHQ data, 1,071 were included in the final analysis (Supplementary Fig. 1). The baseline and demographic characteristics of the participants are presented in Table 1. A total of 220 participants had hypertension, and 276 were taking antihypertensive medications.

**Table 1:** Baseline and demographic characteristics of the participants with and without hypertension

Variables	Total (*N* = 1071)	Hypertension (*n* = 220)	Non-hypertension (*n* = 851)	*P*-value
Demographic characteristics				
Age (year)	54.1 ± 15.1	59.7 ± 13.0	52.7 ± 15.3	<0.001
Sex (Male, n [%])	412 (38.5%)	108 (49.1%)	304 (35.7%)	<0.001
BMI (kg/m^2^)	22.8 ± 3.4	24.1 ± 3.9	22.5 ± 3.2	<0.001
Smoking status (n [%])				<0.05
Current	183 (17.1%)	26 (11.8%)	157 (18.4%)	
Former	202 (18.9%)	58 (26.4%)	144 (16.9%)	
Never	685 (64.0%)	136 (61.8%)	549 (64.5%)	
Drinking status (n [%])				0.24
Current	463 (43.2%)	105 (47.7%)	358 (42.1%)	
Former	52 (4.9%)	12 (5.5%)	40 (4.7%)	
Never	556 (51.9%)	103 (46.8%)	453 (53.2%)	
Exercise frequency [min/week]	54.1 ± 140.6	72 ± 155.1	49.5 ± 136.3	0.09
Nutrients				
Energy [kcal/day]	1848.9 ± 568.2	1879.5 ± 562.7	1841.0 ± 570.0	0.35
Carbohydrate [g/1000 kcal/day]	137.5 ± 19.3	137.0 ± 20.4	137.6 ± 19.0	0.90
[% of energy]	55.0 ± 7.7	54.8 ± 8.2	55.0 ± 7.6	0.90
Fat [g/1000 kcal/day]	27.7 ± 6.2	26.4 ± 6.2	28.0 ± 6.1	<0.001
[% of energy]	24.9 ± 5.5	23.8 ± 5.6	25.2 ± 5.5	<0.001
Protein [g/1000 kcal/day]	37.0 ± 7.2	37.0 ± 8.0	37.0 ± 7.0	0.67
[% of energy]	14.8 ± 2.9	14.8 ± 3.2	14.8 ± 2.8	0.67
Salt (Salt equivalent) [g/day]	10.8 ± 3.6	11.1 ± 3.9	10.7 ± 3.6	0.08
Male (n = 412)	12.1 ± 3.9	12.2 ± 4.2	12.1 ± 3.8	0.71
Female (n = 659)	10.0 ± 3.2	10.1 ± 2.8	10.0 ± 3.2	0.39
Minerals				
Sodium [mg/1000 kcal/day]	2344.5 ± 483.3	2380.9 ± 519.3	2335.1 ± 473.4	0.27
Potassium [mg/1000 kcal/day]	1237.5 ± 342.4	1215.5 ± 367.8	1243.2 ± 335.5	0.21
Calcium [mg/1000 kcal/day]	264.6 ± 90.1	261.3 ± 93.6	265.4 ± 89.2	0.58
Phosphorus [mg/1000 kcal/day]	549.9 ± 113.2	546.5 ± 124.0	550.8 ± 110.2	0.42
Dairy				
Low-fat [g/1000 kcal/day]	17.2 ± 35.2	19.3 ± 39.6	16.7 ± 33.9	0.81
Whole- and high-fat [g/1000 kcal/day]	38.2 ± 44.1	36.0 ± 43.4	38.7 ± 44.2	0.23
Total [g/1000 kcal/day]	55.4 ± 50.9	55.3 ± 51.2	55.5 ± 50.9	0.88
Other food				
Vegetables [g/1000 kcal/day]	112.8 ± 65.8	107.9 ± 69.5	114.1 ± 64.8	0.10
Fruits [g/1000 kcal/day]	28.2 ± 29.3	30.4 ± 32.6	27.6 ± 28.4	0.71
Blood pressure				
Systolic blood pressure [mmHg]	122.1 ± 17.3	145.6 ± 11.7	116.0 ± 12.8	<0.001
Diastolic blood pressure [mmHg]	74.8 ± 11.6	88.4 ± 10.0	71.3 ± 9.1	<0.001

Participants with systolic blood pressures of ≥140 or diastolic blood pressures of ≥90 mmHg were classified as having hypertension

*BMI* body mass index, *baPWV* brachial-ankle pulse wave velocity

### Association of dairy consumption with hypertension, systolic blood pressure, and diastolic blood pressure in all participants

Associations with blood pressure were analyzed using multiple regression models, whereas associations between dairy consumption and hypertension risk were analyzed using logistic regression models. Whole- and high-fat dairy consumption showed significant inverse associations with SBP for all of the models (Table 2). However, no significant relationship was found between whole- and high-fat dairy consumption and DBP or hypertension risk (Table 2 and Supplementary Table 2). Neither low-fat nor total dairy consumption was significantly associated with hypertension risk, SBP, or DBP in any of the models. No significant relationship was observed between dairy intake and the risk of hypertension, even when including all users of antihypertensive medications among those with hypertension (data not shown).

**Table 2:** Association of dairy consumption with systolic and diastolic blood pressure in the overall cohort (*N* = 1071)

	*β*	(95% CI) *SE*	*r*^*2*^	*P*-value
Model 1 (Adjustment factors: Age, Sex, BMI, Medicine intake)				
Systolic blood pressure				
Low-fat dairy products	6.419E-05	(−0.026, 0.026) 0.013	0.244	0.996
Whole- and high-fat dairy products	−0.0213	(−0.042, -0.001) 0.011	0.246	0.044*
Total dairy products	−0.0161	(−0.034, 0.002) 0.009	0.246	0.080
Diastolic blood pressure				
Low-fat dairy products	−0.0035	(−0.022, 0.015) 0.009	0.138	0.708
Whole- and high-fat dairy products	−0.0051	(−0.020, 0.010) 0.008	0.138	0.496
Total dairy products	−0.0056	(−0.018, 0.007) 0.007	0.138	0.393
Model 2 (Adjustment factors: Model 1 + Smoking [current, former, never])				
Systolic blood pressure				
Low-fat dairy products	−0.0010	(−0.027, 0.025) 0.013	0.257	0.940
Whole- and high-fat dairy products	−0.0230	(−0.044, −0.002) 0.011	0.256	0.029*
Total dairy products	−0.0179	(−0.036, 8.79E-05) 0.009	0.255	0.051
Diastolic blood pressure				
Low-fat dairy products	−0.0042	(−0.023, 0.014) 0.009	0.148	0.655
Whole- and high-fat dairy products	−0.0054	(−0.020, 0.009) 0.008	0.148	0.477
Total dairy products	−0.0061	(−0.019, 0.007) 0.007	0.148	0.351
Model 3 (Adjustment factors: Model 2 + Salt, Vegetable, Fruit intake [g/1000 kcal]), Exercise time,				
Drinking [current, former, never]				
Systolic blood pressure				
Low-fat dairy products	0.0004	(−0.026, 0.026) 0.013	0.255	0.974
Whole- and high-fat Dairy Products	−0.0200	(−0.041, 0.001) 0.011	0.258	0.060
Total dairy products	−0.0152	(−0.034, 0.003) 0.009	0.257	0.104
Diastolic blood pressure				
Low-fat dairy products	−0.0021	(−0.021, 0.016) 0.009	0.157	0.823
Whole- and high-fat dairy products	−0.0027	(−0.018, 0.012) 0.008	0.157	0.720
Total dairy products	−0.0031	(−0.016, 0.010) 0.007	0.157	0.637

*β*, partial regression coefficient for each dairy intake. *r*^*2*^, adjusted *r*^*2*^

*CI* confidence interval, *SE* standard error

*P < 0.05

Bold values: significant at *P* < 0.05

To confirm the effect of whole- and high-fat dairy consumption on SBP, SBP was compared between those who did not consume whole- and high-fat dairy products and those who consumed more than the standard daily serving (200 g/day). The average SBP of the former group was 123.9 mmHg, and that of the latter was 116.1 mmHg (Supplementary Fig. 2).

### Association of dairy consumption with hypertension, systolic blood pressure, and diastolic blood pressure in non-users of antihypertensive medicines

To eliminate the influence of antihypertensive medications, we analyzed the participants group who were not using antihypertensive medications. Low-fat dairy consumption was not significantly associated with blood pressure or hypertension risk. However, both whole and high-fat dairy consumption, and total dairy consumption, were inversely associated with SBP and systolic hypertension in all of the models (Tables 3 and 4). When we compared those who did not consume whole- and high-fat dairy products with those who consumed >1 serving of whole- and high-fat dairy products per day, the average SBPs of the former and latter were 120.6 mmHg and 112.0 mmHg, respectively (Supplementary Fig. 3).

**Table 3:** Association of dairy consumption with systolic and diastolic blood pressure in non-users of antihypertensive medications (*n* = 795)

	*β*	(95% CI) *SE*	*r*^*2*^	*P*-value
Model 1 (Adjustment factors: Age, Sex, BMI)				
Systolic blood pressure				
Low-fat dairy products	0.0014	(−0.030, 0.033) 0.016	0.212	0.932
Whole- and high-fat dairy products	−0.0306	(−0.054, −0.007) 0.012	0.219	0.011*
Total dairy products	−0.0232	(−0.044, −0.002) 0.011	0.217	0.029*
Diastolic blood pressure				
Low-fat dairy products	−0.0130	(−0.036, 0.010) 0.012	0.158	0.260
Whole- and high-fat dairy products	−0.0108	(−0.028, 0.006) 0.009	0.158	0.217
Total dairy products	−0.0143	(−0.029, 0.001) 0.008	0.160	0.065
Model 2 (Adjustment factors: Model 1 + Smoking [current, former, never])				
Systolic blood pressure				
Low-fat dairy products	0.0005	(−0.031, 0.032) 0.016	0.220	0.975
Whole- and high-fat dairy products	−0.0326	(−0.056, −0.009) 0.012	0.227	0.007**
Total dairy products	−0.0252	(−0.046, −0.004) 0.011	0.226	0.018*
Diastolic blood pressure				
Low-fat dairy products	−0.0133	(−0.036, 0.009) 0.012	0.166	0.248
Whole- and high-fat dairy products	−0.0111	(−0.028, 0.006) 0.009	0.166	0.206
Total dairy products	−0.0146	(−0.030, 0.001) 0.058	0.168	0.058
Model 3 (Adjustment factors: Model 2 + Salt, Vegetable, Fruit intake [g/1000 kcal], Exercise time, di)				
Drinking [current, former, never]				
Systolic blood pressure				
Low-fat dairy products	0.0019	(−0.029, 0.033) 0.016	0.231	0.906
Whole- and high-fat dairy products	−0.0294	(−0.053, −0.006) 0.012	0.237	0.015*
Total dairy products	−0.0223	(−0.043, −0.001) 0.011	0.236	0.039*
Diastolic blood pressure				
Low-fat dairy products	−0.0106	(−0.033, 0.012) 0.012	0.181	0.357
Whole- and high-fat dairy products	−0.0069	(−0.024, 0.010) 0.009	0.181	0.430
Total dairy products	−0.0103	(−0.026, 0.005) 0.008	0.182	0.186

*β*, partial regression coefficient for each dairy intake. *r*^*2*^, adjusted *r*^*2*^

*CI* confidence interval, *SE* standard error

*P < 0.05, **P < 0.01

Bold values: significant at *P* < 0.05

**Table 4:** Association of dairy consumption with hypertension risk in non-users of antihypertensive medications (*n* = 795)

	OR	(95% CI)	*r*^*2*^	*P*-value
Model 1 (Adjustment factors: Age, Sex, BMI)				
Hypertension (SBP ≥ 140 or DBP ≥ 90)				
Low-fat dairy products	1.0012	(0.9957, 1.0067)	0.102	0.678
Whole- and high-fat dairy products	0.9958	(0.9909, 1.0007)	0.106	0.090
Total dairy products	0.9975	(0.9934, 1.0016)	0.104	0.232
Systolic hypertension (SBP ≥ 140)				
Low-fat dairy products	1.0004	(0.9943, 1.0066)	0.107	0.900
Whole- and high-fat dairy products	0.9927	(0.9868, 0.9986)	0.118	0.016*
Total dairy products	0.9948	(0.9898, 0.9998)	0.115	0.041*
Diastolic hypertension (DBP ≥ 90)				
Low-fat dairy products	1.0001	(0.9935, 1.0068)	0.079	0.969
Whole- and high-fat dairy products	0.9968	(0.9912, 1.0024)	0.082	0.263
Total dairy products	0.9977	(0.9929, 1.0025)	0.081	0.345
Model 2 (Adjustment factors: Model1 + Smoking [current, former, never])				
Hypertension (SBP ≥ 140 or DBP ≥ 90)				
Low-fat dairy products	1.0010	(0.9955, 1.0066)	0.109	0.721
Whole- and high-fat dairy products	0.9955	(0.9905, 1.0004)	0.113	0.071
Total dairy products	0.9971	(0.9930, 1.0013)	0.111	0.183
Systolic hypertension (SBP ≥ 140)				
Low-fat dairy products	1.0003	(0.9942, 1.0065)	0.108	0.916
Whole- and high-fat dairy products	0.9925	(0.9865, 0.9984)	0.120	0.013*
Total dairy products	0.9946	(0.9895, 0.9996)	0.117	0.034*
Diastolic hypertension (DBP ≥ 90)				
Low-fat dairy products	0.9999	(0.9932, 1.0067)	0.092	0.978
Whole- and high-fat dairy products	0.9966	(0.9909, 1.0023)	0.095	0.240
Total dairy products	0.9974	(0.9925, 1.0023)	0.094	0.297
Model 3 (Adjustment factors: Model2 + Salt, Vegetable, Fruit intake [g/1000 kcal]), Exercise time,				
Drinking [current, former, never]				
Hypertension (SBP ≥ 140 or DBP ≥ 90)				
Low-fat dairy products	1.0008	(0.9951, 1.0065)	0.127	0.795
Whole- and high-fat dairy products	0.9955	(0.9905, 1.0006)	0.131	0.085
Total dairy products	0.9971	(0.9927, 1.0014)	0.129	0.187
Systolic hypertension (SBP ≥ 140)				
Low-fat dairy products	0.9999	(0.9934, 1.0063)	0.132	0.967
Whole- and high-fat dairy products	0.9924	(0.9864, 0.9985)	0.144	0.015**
Total dairy products	0.9942	(0.9890, 0.9994)	0.141	0.030*
Diastolic hypertension (DBP ≥ 90)				
Low-fat dairy products	1.0000	(0.9932, 1.0069)	0.109	0.998
Whole- and high-fat dairy products	0.9973	(0.9914, 1.0033)	0.110	0.376
Total dairy products	0.9980	(0.9930, 1.0031)	0.110	0.447

*OR* odds ratio for each dairy intake, *CI* confidence interval, *SBP* systolic blood pressure, *DBP* diastolic blood pressure

*r*^*2*^, pseudo *r*^*2*^

*P < 0.05, **P < 0.01

Bold values: significant at *P* < 0.05

### Associations between blood biomarkers, dairy consumption, and systolic blood pressure

**Fig. 1:**
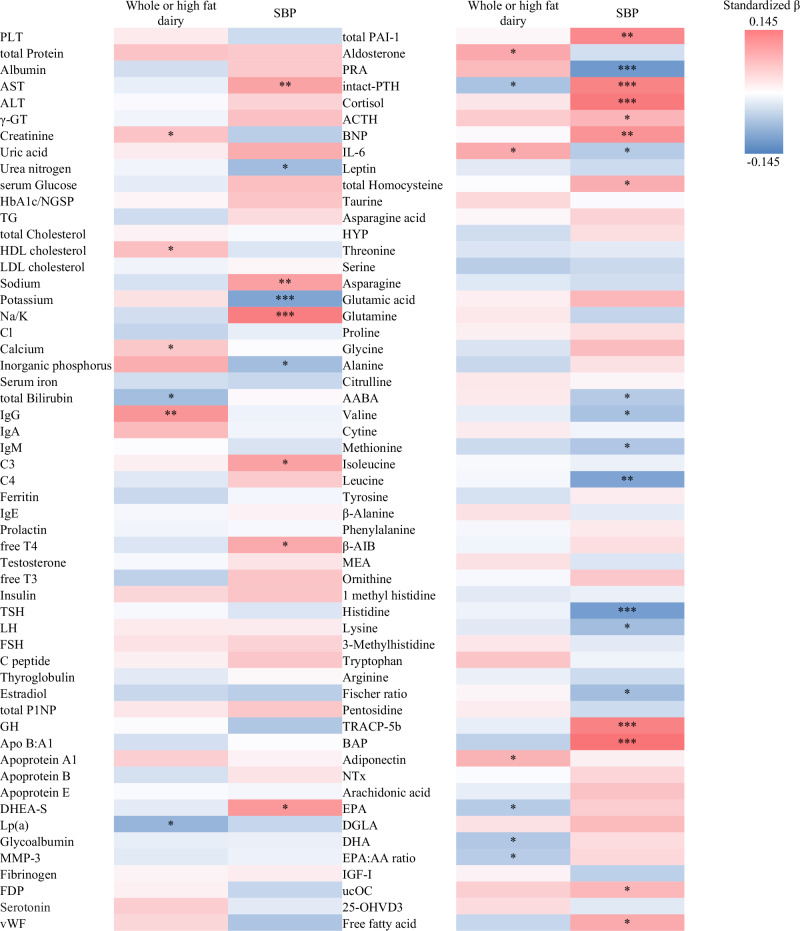
Heat map for standardized partial regression coefficients between blood biomarkers and dairy consumption or systolic blood pressure. Standardized β, standardized partial regression coefficients; SBP, systolic blood pressure. Other abbreviations are listed in Supplementary Table 1. * *P* < 0.05, ** *P* < 0.01, *** *P* < 0.001

Various blood biomarkers were measured to identify the factors associated with dairy consumption and lower SBP in non-users of antihypertensive medications. Of the 110 total biomarkers studied (Supplementary Table 1), 13 were significantly associated with whole- and high-fat dairy consumption. Seven had a direct association, while six had an inverse association. A total of 28 biomarkers were significantly associated with SBP—16 with direct associations and 12 with inverse ones. Among these, inorganic phosphorus, intact parathyroid hormone, and interleukin-6 (IL-6) were associated with both whole- and high-fat dairy consumption, as well as SBP (Fig. 1, Table 5). Inorganic phosphorus and IL-6 were directly associated with whole- and high-fat dairy consumption, and inversely associated with SBP. Whole intact parathyroid hormone was inversely associated with whole- and high-fat dairy consumption, and directly associated with SBP.

**Table 5:** The associations of biomarkers (inorganic phosphorus, PTH-intact, and IL-6) with whole- and high-fat dairy consumption, as well as systolic blood pressure in non-users of antihypertensive medications

	*β*	(95% CI) *SE*	*r*^*2*^	*P*-value
**(Response variable)**	**Whole- and high-fat dairy products (explanatory variable)**			
Inorganic phosphorus (mg/dL)	0.0008	(0.000, 0.001) 0.000	0.155	0.001**
Intact-PTH (pg/mL)	−0.0293	(−0.057, −0.002) 0.014	0.033	0.037*
IL-6 (pg/mL)	0.0086	(0.001, 0.017) 0.004	0.018	0.031*
**(Explanatory variable)**	**Systolic blood pressure (response variable)**			
Inorganic phosphorus (mg/dL)	−2.9856	(−5.551, −0.420) 1.307	0.236	0.023*
Intact-PTH (pg/mL)	0.1143	(0.054, 0.174) 0.031	0.245	<0.001***
IL-6 (pg/mL)	−0.2303	(−0.449, −0.012) 0.111	0.235	0.039*

*β*, partial regression coefficient for each biomarker. *r*^*2*^, adjusted *r*^*2*^

All models were adjusted for age, sex, BMI, smoking status, salt, vegetable, and fruit intake, exercise time, and drinking habits

*Intact-PTH* intact parathyroid hormone, *IL-6* interleukin-6, *CI* confidence interval, *SE*, standard error

*P < 0.05, **P < 0.01, ***P < 0.001

Bold values: significant at *P* < 0.05

Among the all participants, only inorganic phosphorus and intact parathyroid hormone were significantly associated with both whole- and high-fat dairy consumption, as well as SBP. IL-6 was also significantly associated with dairy consumption but did not show a significant relationship with SBP (*P* = 0.052). The directions of the associations were the same as those observed in the analysis for non-users of antihypertensive medications (Supplementary Table 3).

### Association between mineral intake and systolic blood pressure

Previous studies indicate that minerals found in dairy products may help lower blood pressure [15]. Our analysis of blood markers also suggested a relationship between phosphorus and blood pressure. Therefore, we examined the associations between mineral intake and SBP. We found that intakes of potassium, calcium, and phosphorus—minerals abundant in dairy products—were inversely associated with SBP (Supplementary Table 4).

## Discussion

In this cross-sectional analysis, we found that whole- and high-fat dairy consumption was inversely associated with SBP, in a population-based study of community-dwelling healthy Japanese adults. In those participants not taking antihypertensive medications, whole- and high-fat, and total dairy consumption were all inversely associated with SBP and systolic hypertension risk, while there was no association between dairy consumption and DBP. Only three out of the 110 blood biomarkers we analyzed were significantly associated with both whole- and high-fat dairy consumption, as well as SBP. The mean salt intake of the study participants exceeded the WHO’s recommendation of 5 g per day, as well as the average Japanese intake of 10.9 g and 9.3 g for males and females, respectively [31, 32].

Conversely, another study reported that low-fat dairy products were more beneficial for lowering blood pressure than whole- or high-fat ones [33]. In our study, 64.2% of the participants reported no consumption of low-fat dairy products, and the mean low-fat dairy intake was 17.2 g/1000 kcal—4 to 5 times lower than the 92.7 g/1000 kcal calculated in that study. Distinctive patterns of dairy intake may result in various associations with blood pressure and dairy fat levels. Indeed, a healthy dietary pattern, as defined in one cross-sectional study of middle-aged Japanese individuals, contains milk and whole-fat yogurt [34]. In another longitudinal study conducted across different regions of Japan, the participants who did not consume dairy products daily had a higher risk of hypertension over a 10-year follow-up period, irrespective of fat levels [35]. This highlighted the protective effects of dietary calcium against a number of cardiovascular diseases [36, 37], suggesting that it plays a promising role in the relationship between dairy consumption and hypertension. Considering that calcium content varies by the type of dairy consumed, along with different levels of absorption in the food matrix [38, 39], further studies are warranted to determine the associations between different dairy products and hypertension in Japanese populations.

### Factors associated with dairy consumption and blood pressure

Among the numerous blood biomarkers, only three had a significant relationship with both whole- and high-fat dairy consumption, as well as SBP. In particular, inorganic phosphorus and intact parathyroid hormone levels were relatively well correlated (Table 5). Dairy products are rich in micronutrients such as vitamins, calcium, and inorganic phosphorus [11]. Our finding that whole- and high-fat dairy consumption was directly associated with inorganic phosphorus blood concentrations suggests that dairy consumption increases the levels of blood inorganic phosphorus. An international cross-sectional study showed that phosphorus intake was inversely associated with blood pressure in middle-aged individuals, independent of other nutrients [14]. Our study builds on the results of that report in a population-based setting comprising Japanese adults.

Phosphorus metabolism is regulated through a number of complex mechanisms, as it plays crucial roles in maintaining biological processes such as energy metabolism, cellular signaling, and bone mineralization [40]. Parathyroid hormone is a key regulator of mineral metabolism, including phosphate [41]. It promotes bone resorption and suppresses phosphorus reabsorption in the proximal tubules, raising serum phosphorus levels [42]. Several studies have reported a relationship between high plasma parathyroid hormone levels and elevated blood pressure [43, 44]. In a Korean population-based study, a positive association was found between serum parathyroid hormone level and SBP along with the prevalence of atrial fibrillation [43]. Parathyroid hormone also plays a role in the regulation of immune responses. Elevated IL-6 levels have been observed in patients with hyperparathyroidism [45]. Taken together, the increase in blood inorganic phosphorus levels caused by high dairy intake may reduce the secretion of parathyroid hormone, leading to the reduction of SBP and IL-6 levels. Although phosphorus intake comes from sources other than dairy products, it was found to be inversely associated with SBP (Supplementary Table 4).

Dairy products, which are rich in phosphorus and calcium, may contribute to lower blood pressure [33], which aligns with our findings. Various mechanisms have been proposed to describe how parathyroid hormone affects blood pressure, and elevated parathyroid hormone levels have been reported to predict long-term vascular stiffness [44, 46]. This suggests that whole- and high-fat dairy products may prevent hypertension in the long-term by maintaining elasticity in blood vessels.

### Potassium metabolism

The dietary sodium-to-potassium ratio is positively associated with blood pressure [47]. Potassium intake was also significantly associated with blood pressure in this study (Supplementary Table 4). As dairy products are rich sources of potassium, they are inversely associated with the dietary sodium-to-potassium ratio [47]. These findings suggest that dairy products may suppress blood pressure through an abundance of potassium, in addition to phosphorus. A recent meta-analysis indicated that the urinary sodium-to-potassium ratio is associated with lower blood pressure in adults [48]. Milk and dairy products have been reported to be associated with the urinary sodium-to-potassium ratio in Japanese male adolescents, along with socioeconomic status (independently of fruit and dairy intakes) [49]. However, we found no association between sodium-to-potassium ratio or blood potassium concentration and blood pressure. Considering that sodium and potassium levels are strictly regulated in blood homeostasis, this result does not appear to contradict the above-mentioned findings.

### Strengths and limitations

The strengths of this study include its cross-sectional analysis of blood pressure in a community-based population with high salt and low dairy diets. We examined the possible relationship between dairy consumption and blood pressure in the high salt and low dairy dietary pattern that is often observed not only in Japan but also in other Asian populations [50, 51]. Our comprehensive analysis of blood biomarkers to their associations with dairy consumption and blood pressure represents another major strength, as the IHPP included a series of blood biomarker measurements performed during comprehensive medical checkups.

Nevertheless, this study had some limitations. First, the BDHQ does not distinguish between different dairy products, such as milk or yogurt, and lacks data on cheese and other dairy-based foods. We were thus unable to identify any associations related to different dairy products. Additionally, some dietary minerals can be assessed using 24-h dietary recalls, which are considered a gold-standard method from the viewpoint of validity. Second, causality was not clarified, owing to the nature of cross-sectional analyses. Although phosphorus metabolism has been associated with dairy consumption and blood pressure, further studies are warranted to confirm this hypothesis for this population. Finally, the IHPP targeted a rural population with a high mean age that included a few young adults, resulting in a potential selection bias.

### Perspective of Asia

Japanese have lower dairy intake and higher salt intake than Westerners. Although previous studies have not found a consistent effect of dairy intake on blood pressure or prevention of hypertension [17, 18], it is possible that dairy intake have greater impact on blood pressure for those with lower dairy intake and higher salt intake such as Asian populations [50, 51].

## Conclusion

Dairy consumption was found to be partially inversely associated with SBP in a Japanese population with high salt and low dairy intake. Higher whole- and high-fat dairy consumption and higher total dairy consumption were associated with lower blood pressure and lower risk of systolic hypertension among those not using antihypertensive medications. Phosphorus metabolism is likely involved in the relationship between dairy consumption and blood pressure. These findings offer promising opportunities for enhancing blood pressure management and underscore the potential benefits of dairy products.

## Supplementary information

Supplementary Table 1

Supplementary Table 2

Supplementary Table 3

Supplementary Table 4

Supplementary Fig. 1

Supplementary Fig. 2

Supplementary Fig. 3

## Data Availability

Data, including analytical codes, cannot be shared publicly due to ethical concerns. Data are available from the Hirosaki University COI Institutional Data Access/Ethics Committee (contact via e-mail: https://coi@hirosaki-u.ac.jp) for researchers who meet the criteria for data access. Researchers must be approved by the research ethics review board of the organization of their affiliation.
