# Supplementary material for: Dairy consumption has a partial inverse association with systolic blood pressure and hypertension in populations with high salt and low dairy diets: cross-sectional data analysis from the Iwaki Health Promotion Project
Source: Hypertens Res. 2025 Jan 22;48(4):1409–21. doi: 10.1038/s41440-024-02088-6 (PMC11972955; doi:10.1038/s41440-024-02088-6)
Supplement: Supplementary file 1 — Supplementary Table 1 [file 41440_2024_2088_MOESM1_ESM.docx]

Supplementary Table 1. List of measured biomarkers

| **Measurements** | **Abbreviation** | **Method** |
| --- | --- | --- |
| Platelet | PLT | Flow cytometry |
| Total Protein |  | Biuret |
| Albumin |  | Improvement Bromocresol purple |
| Aspartate aminotransferase | AST | JSCC Standardization method |
| Alanine transaminase | ALT | JSCC Standardization method |
| γ-glutamyl transferase | γ-GT | JSCC Standardization method |
| Creatinine |  | Enzymatic method |
| Uric acid |  | Enzymatic method |
| Urea nitrogen |  | Urease |
| Serum Glucose |  | Enzymatic method (GK-G6PD) |
| Hemoglobin A1c (National Glycohemoglobin Standardization Program) | HbA1c/NGSP | Enzymatic method |
| Triglyceride | TG | Enzymatic method |
| total Cholesterol |  | Enzymatic method |
| HDL cholesterol |  | Enzymatic method |
| LDL cholesterol |  | Enzymatic method |
| Sodium |  | Electrode method |
| Potassium |  | Electrode method |
| Sodium potassium ratio | Na/K |  |
| Chloride | Cl | Electrode method |
| Calcium |  | Arsenazo Ⅲ method |
| Inorganic phosphorus |  | Enzymatic method |
| Serum iron |  | Colorimetric method |
| Total Bilirubin |  | Enzymatic method |
| Immunoglobulin G | IgG | Turbidimetric immunoassay method |
| Immunoglobulin A | IgA | Turbidimetric immunoassay method |
| Immunoglobulin M | IgM | Turbidimetric immunoassay method |
| Complement component 3 | C3 | Turbidimetric immunoassay method |
| Complement component 4 | C4 | Turbidimetric immunoassay method |
| Ferritin |  | Chemiluminescent immunoassay |
| Immunoglobulin E | IgE | Fluorescent enzyme immunoassay |
| Prolactin |  | Chemiluminescent immunoassay |
| Free Triiodothyronine 4 | free T4 | Chemiluminescent immunoassay |
| Testosterone |  | Chemiluminescent immunoassay |
| Free Triiodothyronine 3 | free T3 | Chemiluminescent immunoassay |
| Insulin |  | Chemiluminescent immunoassay |
| Thyroid-stimulating hormone | TSH | Chemiluminescent immunoassay |
| Luteinizing hormone | LH | Chemiluminescent immunoassay |
| Follicle-stimulating hormone | FSH | Chemiluminescent immunoassay |
| C peptide |  | Chemiluminescent immunoassay |
| Thyroglobulin |  | Electro chemiluminescence immunoassay |
| Estradiol |  | Chemiluminescent immunoassay |
| Total plasminogen activator inhibitor 1 | total P1NP | Electro chemiluminescence immunoassay |
| Growth hormone | GH | Electro chemiluminescence immunoassay |
| Apoprotein B:A1 ratio |  |  |
| Apoprotein A1 |  | Turbidimetric immunoassay method |
| Apoprotein B |  | Turbidimetric immunoassay method |
| Apoprotein E |  | Turbidimetric immunoassay method |
| Dehydroepiandrosterone sulfate | DHEA-S | Chemiluminescent enzyme immunoassay |
| Lipoprotein (a) | Lp(a) | Turbidimetric immunoassay method |
| Glycoalbumin |  | Enzymatic method |
| Matrix metalloproteinase-3 | MMP-3 | Latex agglutination |
| Fibrinogen |  | Thrombin method |
| Fibrinogen/fibrin degradation products | FDP | Latex photometric immunoassay |
| Serotonin |  | High-performance liquid chromatography |
| von Willebrand factor | vWF | Platelet aggregation |
| Total Plasminogen activator inhibitor 1 | total PAI-1 | Latex agglutination |
| Aldosterone |  | Radioimmunoassay |
| Plasma renin activity | PRA | Radioimmunoassay |
| Intact Parathyroid hormone | intact-PTH | Electro chemiluminescence immunoassay |
| Cortisol |  | Chemiluminescent enzyme immunoassay |
| Adrenocorticotropic hormone | ACTH | Electro chemiluminescence immunoassay |
| Brain natriuretic peptide | BNP | Chemiluminescent enzyme immunoassay |
| Interleukin-6 | IL-6 | Chemiluminescent enzyme immunoassay |
| Leptin |  | Radioimmunoassay |
| Total Homocysteine |  | Liquid chromatography-tandem mass spectrometry |
| Taurine |  | High-performance liquid chromatography |
| Asparagine acid |  | High-performance liquid chromatography |
| Hydroxyproline | HYP | High-performance liquid chromatography |
| Threonine |  | High-performance liquid chromatography |
| Serine |  | High-performance liquid chromatography |
| Asparagine |  | High-performance liquid chromatography |
| Glutamic acid |  | High-performance liquid chromatography |
| Glutamine |  | High-performance liquid chromatography |
| Proline |  | High-performance liquid chromatography |
| Glycine |  | High-performance liquid chromatography |
| Alanine |  | High-performance liquid chromatography |
| Citrulline |  | High-performance liquid chromatography |
| α-Aminobutyric acid | AABA | High-performance liquid chromatography |
| Valine |  | High-performance liquid chromatography |
| Cystine |  | High-performance liquid chromatography |
| Methionine |  | High-performance liquid chromatography |
| Isoleucine |  | High-performance liquid chromatography |
| Leucine |  | High-performance liquid chromatography |
| Tyrosine |  | High-performance liquid chromatography |
| β-Alanine |  | High-performance liquid chromatography |
| Phenylalanine |  | High-performance liquid chromatography |
| β‐Aminoisobutyric acid | β‐AIB | High-performance liquid chromatography |
| Monoethanolamine | MEA | High-performance liquid chromatography |
| Ornithine |  | High-performance liquid chromatography |
| 1-Methyl histidine |  | High-performance liquid chromatography |
| Histidine |  | High-performance liquid chromatography |
| Lysine |  | High-performance liquid chromatography |
| 3-Methyl histidine |  | High-performance liquid chromatography |
| Tryptophan |  | High-performance liquid chromatography |
| Arginine |  | High-performance liquid chromatography |
| Fischer ratio |  |  |
| Pentosidine |  | High-performance liquid chromatography |
| Tartrate-resistant acid phosphatase 5b | TRACP-5b | Enzyme immunoassay |
| Bone Specific Alkaline Phosphatase | BAP | Chemiluminescent enzyme immunoassay |
| Adiponectin |  | Latex agglutination |
| Type I collagen cross-linked N-telopeptides | NTx | Enzyme immunoassay |
| Arachidonic acid |  | Liquid chromatography-tandem mass spectrometry |
| Eicosapentaenoic acid | EPA | Liquid chromatography-tandem mass spectrometry |
| Dihomo-gamma-linolenic acid | DGLA | Liquid chromatography-tandem mass spectrometry |
| Docosahexaenoic acid | DHA | Liquid chromatography-tandem mass spectrometry |
| Eicosapentaenoic acid: Arachidonic acid ratio | EPA:AA ratio |  |
| Insulin-like Growth Factor I | IGF-I | Electro chemiluminescence immunoassay |
| undercarboxylated osteocalcin | ucOC | Immuno radio metric assay |
| 25-hydroxy vitamin D3 | 25-OHVD3 | Electro chemiluminescence immunoassay |
| Free fatty acid |  | Enzymatic method |
