# Supplementary material for: Dairy consumption has a partial inverse association with systolic blood pressure and hypertension in populations with high salt and low dairy diets: cross-sectional data analysis from the Iwaki Health Promotion Project
Source: Hypertens Res. 2025 Jan 22;48(4):1409–21. doi: 10.1038/s41440-024-02088-6 (PMC11972955; doi:10.1038/s41440-024-02088-6)
Supplement: Supplementary file 2 — Supplementary Table 2 [file 41440_2024_2088_MOESM2_ESM.docx]

Supplementary Table 2. Association of dairy consumption with hypertension risk (N=1071)

|  | **OR** | | **(95% CI)** | | | | ***r^2^*** | | | ***P*-value** | | | | | | | |  |
| --- | --- | --- | --- | --- | --- | --- | --- | --- | --- | --- | --- | --- | --- | --- | --- | --- | --- | --- |
| Model 1 (Adjustment factors: Age, Sex, BMI, Medicine intake) | | | | | | | | | | | | |  |  |  |  |  |  |
| Hypertension (SBP≥140 or DBP≥90) |  |  | | |  | | |  | | | |  |  |  |  |  |  |  |
| Low-fat dairy products | 1.0001 | | (0.9959–1.0044) | | | | 0.079 | | | 0.950 | | | | | | |  |  |
| Whole- and high-fat dairy products | 0.9989 | | (0.9953–1.0026) | | | | 0.080 | | | 0.564 | | | | | | |  |  |
| Total dairy products | 0.9993 | | (0.9961–1.0024) | | | | 0.079 | | | 0.651 | | | | | | |  |  |
| Systolic hypertension (SBP≥140) |  |  | | |  | | |  | | | |  |  |  |  |  |  |  |
| Low-fat dairy products | 0.9999 | | (0.9952–1.0045) | | | | 0.088 | | | 0.954 | | | | | | |  |  |
| Whole- and high-fat dairy products | 0.9978 | | (0.9937–1.0019) | | | | 0.089 | | | 0.287 | | | | | | |  |  |
| Total dairy products | 0.9982 | | (0.9947–1.0018) | | | | 0.089 | | | 0.332 | | | | | | |  |  |
| Diastolic hypertension (DBP≥90) |  |  | | |  | | |  | | | |  |  |  |  |  |  |  |
| Low-fat dairy products | 1.0007 | | (0.9953–1.0062) | | | | 0.055 | | | 0.794 | | | | | | |  | |
| Whole- and high-fat dairy products | 0.9982 | | (0.9935–1.0029) | | | | 0.056 | | | 0.460 | | | | | | |  | |
| Total dairy products | 0.9991 | | (0.9952–1.0030) | | | | 0.055 | | | 0.652 | | | | | | |  | |
| Model 2 (Adjustment factors: Model 1 + Smoking [current, former, never]) | | | | | | | | | | | | | |  |  |  |  |  |
| Hypertension (SBP≥140 or DBP≥90) |  |  | | |  | | |  | | | |  | |  |  |  |  |  |
| Low-fat dairy products | 0.9999 | | (0.9956–1.0043) | | | | 0.086 | | | 0.980 | | | | | | |  |  |
| Whole- and high-fat dairy products | 0.9988 | | (0.9952–1.0025) | | | | 0.086 | | | 0.533 | | | | | | |  |  |
| Total dairy products | 0.9991 | | (0.9959–1.0023) | | | | 0.086 | | | 0.578 | | | | | | |  |  |
| Systolic hypertension (SBP≥140) |  |  | | |  | | |  | | | |  |  |  |  |  |  |  |
| Low-fat dairy products | 0.9998 | | (0.9951–1.0044) | | | | 0.091 | | | 0.917 | | | | | | |  |  |
| Whole- and high-fat dairy products | 0.9977 | | (0.9936–1.0018) | | | | 0.092 | | | 0.264 | | | | | | |  |  |
| Total dairy products | 0.9981 | | (0.9945–1.0017) | | | | 0.092 | | | 0.293 | | | | | | |  |  |
| Diastolic hypertension (DBP≥90) |  |  | | | |  | | |  | | | | | |  |  |  |  |
| Low-fat dairy products | 1.0005 | | (0.9950–1.0060) | | | | 0.066 | | | 0.869 | | | | | | |  |  |
| Whole- and high-fat dairy products | 0.9981 | | (0.9934–1.0029) | | | | 0.066 | | | 0.436 | | | | | | |  |  |
| Total dairy products | 0.9989 | | (0.9949–1.0029) | | | | 0.066 | | | 0.581 | | | | | | |  |  |
| Model 3 (Adjustment factors: Model 2 + Salt, Vegetable, and Fruit intake [g/1000 kcal], Exercise | | | | | | | | | | | | | | | | |  |  |
| time, Drinking [current, former, never]) | | | | |  | | |  | | | |  |  |  |  |  |  |  |
| Hypertension (SBP≥140 or DBP≥90) |  |  | | |  | | |  | | | |  |  |  |  |  |  |  |
| Low-fat dairy products | 1.0000 | | (0.9956–1.0044) | | | | 0.09 | | | 0.996 | | | | | | |  |  |
| Whole- and high-fat dairy products | 0.9987 | | (0.9950–1.0024) | | | | 0.09 | | | 0.492 | | | | | | |  |  |
| Total dairy products | 0.9990 | | (0.9958–1.0023) | | | | 0.09 | | | 0.557 | | | | | | |  |  |
| Systolic hypertension (SBP≥140) |  |  | |  | | |  | | |  |  |  |  |  |  |  |  |  |
| Low-fat dairy products | 0.9999 | | (0.9952–1.0047) | | | | 0.096 | | | 0.97 | | | | | | |  |  |
| Whole- and high-fat dairy products | 0.9977 | | (0.9935–1.0019) | | | | 0.098 | | | 0.275 | | | | | | |  |  |
| Total dairy products | 0.9981 | | (0.9944–1.0017) | | | | 0.098 | | | 0.304 | | | | | | |  |  |
| Diastolic hypertension (DBP≥90) |  |  | |  | | |  | | |  |  |  |  |  |  |  |  |  |
| Low-fat dairy products | 1.0009 | | (0.9953–1.0064) | | | | 0.073 | | | 0.758 | | | | | | |  |  |
| Whole- and high-fat dairy products | 0.9986 | | (0.9938–1.0035) | | | | 0.073 | | | 0.574 | | | | | | |  |  |
| Total dairy products | 0.9994 | | (0.9954–1.0035) | | | | 0.073 | | | 0.792 | | | | | | |  |  |

Abbreviations: OR, odds ratio for each dairy intake; CI, confidence interval; SBP, systolic blood pressure; DBP, diastolic blood pressure.

*r^2^*, pseudo *r^2^*.
