# Supplementary material for: Dairy consumption has a partial inverse association with systolic blood pressure and hypertension in populations with high salt and low dairy diets: cross-sectional data analysis from the Iwaki Health Promotion Project
Source: Hypertens Res. 2025 Jan 22;48(4):1409–21. doi: 10.1038/s41440-024-02088-6 (PMC11972955; doi:10.1038/s41440-024-02088-6)
Supplement: Supplementary file 3 — Supplementary Table 3 [file 41440_2024_2088_MOESM3_ESM.docx]

Supplementary Table 3. The associations of biomarkers (inorganic phosphorus, PTH-intact, and IL-6) with whole- and high-fat dairy consumption, as well as systolic blood pressure

|  | **β** | **(95% CI) *SE*** | ***r^2^*** | ***P*-value** | |
| --- | --- | --- | --- | --- | --- |
|  | **Whole- and high-fat dairy products** | | | | |
| **(Response variable)** | **(explanatory variable)** | | | | |
| Inorganic phosphorus (mg/dL) | 0.0009 | (0.000, 0.001) 0.000 | 0.165 | **0.001** | ** |
| Intact-PTH (pg/mL) | -0.0409 | (-0.074, -0.008) 0.017 | 0.021 | **0.015** | * |
| IL-6 (pg/mL) | 0.0060 | (0.000, 0.012) 0.003 | 0.019 | 0.052 |  |
| **(Explanatory variable)** | **Systolic blood pressure (response variable)** | | | | |
| Inorganic phosphorus (mg/dL) | -2.9451 | (-5.165, -0.725) 1.131 | 0.260 | **0.009** | ** |
| Intact-PTH (pg/mL) | 0.0481 | (0.010, 0.086) 0.019 | 0.259 | **0.014** | * |
| IL-6 (pg/mL) | -0.2346 | (-0.446, -0.023) 0.108 | 0.256 | **0.029** | * |

Abbreviations: Intact-PTH, intact parathyroid hormone; IL-6, interleukin-6; CI, confidence interval; *SE*, standard error.

β, partial regression coefficient for each biomarker. *r^2^*, adjusted *r^2^*. * *P*<0.05, ** *P*<0.01.
All models were adjusted for age, sex, BMI, antihypertensive medication use, smoking status, salt, vegetable and fruit intake, exercise time, and drinking habits.
