# Supplementary material for: Dairy consumption has a partial inverse association with systolic blood pressure and hypertension in populations with high salt and low dairy diets: cross-sectional data analysis from the Iwaki Health Promotion Project
Source: Hypertens Res. 2025 Jan 22;48(4):1409–21. doi: 10.1038/s41440-024-02088-6 (PMC11972955; doi:10.1038/s41440-024-02088-6)
Supplement: Supplementary file 4 — Supplementary Table 4 [file 41440_2024_2088_MOESM4_ESM.docx]

Supplementary Table 4. Association of mineral intakes with systolic blood pressure

| (mg/1000 kcal) | **β** | **(95% CI) *SE*** | ***r^2^*** | ***P*-value** |  |  |  |
| --- | --- | --- | --- | --- | --- | --- | --- |
| Overall (N=1071) | Adjustment factors: Age, Sex, BMI, Medicine intake, Smoking [current, former, never], Salt intake [g/1000 kcal], Exercise time, Drinking [current, former, never] | | | | | | |
| Potassium (K) | -0.0051 | (-0.008, -0.002) 0.002 | 0.261 | **0.001** | ** |  |  |
| Calcium (Ca) | -0.0167 | (-0.028, -0.005) 0.006 | 0.259 | **0.005** | ** |  |  |
| Phosphorus (P) | -0.0140 | (-0.024, -0.004) 0.005 | 0.259 | **0.006** | ** |  |  |
| Non-users of antihypertensive medicines (n=795) | Adjustment factors: Age, Sex, BMI, Smoking [current, former, never], Salt intake [g/1000 kcal], Exercise time, Drinking [current, former, never] | | | | | | |
| Potassium (K) | -0.0061 | (-0.010, -0.003) 0.002 | 0.238 | **0.001** | ** |  |  |
| Calcium (Ca) | -0.0207 | (-0.034, -0.007) 0.007 | 0.235 | **0.003** | ** |  |  |
| Phosphorus (P) | -0.0167 | (-0.029, -0.005) 0.006 | 0.234 | **0.006** | ** |  |  |

Abbreviations: CI, confidence interval; *SE*, standard error.
β, partial regression coefficient for each dairy intake. *r^2^*, adjusted *r^2^*. ** *P*<0.01.
