# Supplementary material for: Dairy consumption has a partial inverse association with systolic blood pressure and hypertension in populations with high salt and low dairy diets: cross-sectional data analysis from the Iwaki Health Promotion Project
Source: Hypertens Res. 2025 Jan 22;48(4):1409–21. doi: 10.1038/s41440-024-02088-6 (PMC11972955; doi:10.1038/s41440-024-02088-6)
Supplement: Supplementary file 5 — Supplementary Fig. 1 [file 41440_2024_2088_MOESM5_ESM.docx]

Supplementary Figure 1. Flowchart of participant recruitment and grouping


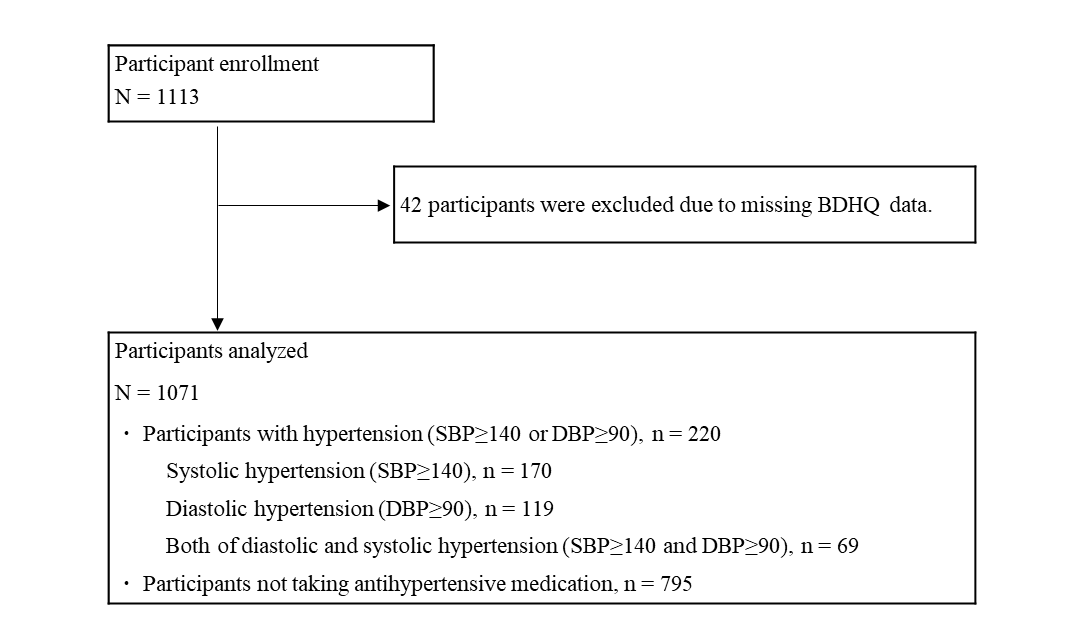


Abbreviations: BDHQ, Brief-type self-administered Diet History Questionnaire; SBP, systolic blood pressure; DBP, diastolic blood pressure.
