# Supplementary material for: Dairy consumption has a partial inverse association with systolic blood pressure and hypertension in populations with high salt and low dairy diets: cross-sectional data analysis from the Iwaki Health Promotion Project
Source: Hypertens Res. 2025 Jan 22;48(4):1409–21. doi: 10.1038/s41440-024-02088-6 (PMC11972955; doi:10.1038/s41440-024-02088-6)
Supplement: Supplementary file 6 — Supplementary Fig. 2 [file 41440_2024_2088_MOESM6_ESM.docx]

Supplementary Figure 2. Comparison of systolic blood pressure by whole- and high-fat dairy product consumption


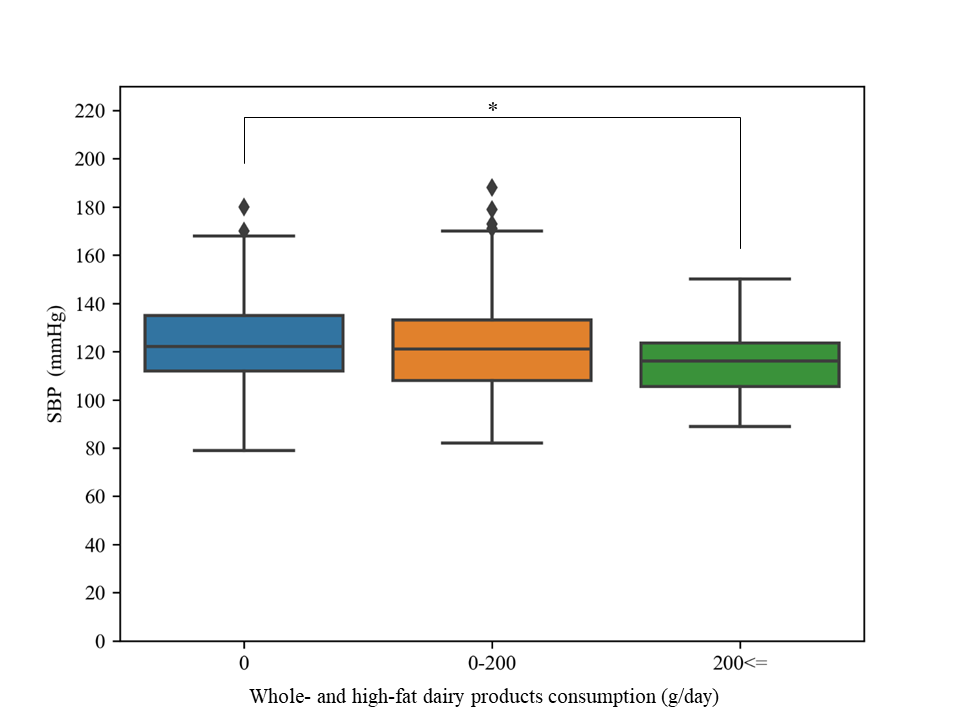


Abbreviations: SBP, systolic blood pressure.

Participants were grouped by whole- and high-fat dairy consumption (g/day): 0, >0, <200, and ≥200; N=309, 719, and 43, respectively. A Steel-Dwass test was performed. * *P*<0.05.
